# Bimodal Ultrasound and X-ray Bioimaging Properties of Particulate Calcium Fluoride Biomaterial

**DOI:** 10.3390/molecules26185447

**Published:** 2021-09-07

**Authors:** Cristhian Marcelo Chingo Aimacaña, Kevin O. Pila, Dilan A. Quinchiguango Perez, Alexis Debut, Mohamed F. Attia, Ralph Santos-Oliveira, Daniel C. Whitehead, Carlos Reinoso, Frank Alexis, Si Amar Dahoumane

**Affiliations:** 1 School of Physical Sciences and Nanotechnology, Yachay Tech University, Urcuquí 100650, Ecuador; cristhian.chingo@yachaytech.edu.ec (C.M.C.A.); creinoso@yachaytech.edu.ec (C.R.); 2 School of Biological Sciences and Engineering, Yachay Tech University, Urcuquí 100650, Ecuador; kevin.pila@yachaytech.edu.ec (K.O.P.); dilan.quinchiguango@yachaytech.edu.ec (D.A.Q.P.); 3 Center of Nanoscience and Nanotechnology, Universidad de las Fuerzas Armadas ESPE, Sangolquí 170501, Ecuador; apdebut@espe.edu.ec; 4 Center for Nanotechnology in Drug Delivery and Division of Pharmaco-engineering and Molecular Pharmaceutics, Eshelman School of Pharmacy, University of North Carolina at Chapel Hill, Chapel Hill, NC 27599, USA; mattia@email.unc.edu; 5 Laboratory of Nanoradiopharmacy and Synthesis of Novel Radiopharmaceuticals, Nuclear Engineering Institute, Brazilian Nuclear Energy Commission, Rio de Janeiro 21941-906, Brazil; presidenciaradiofarmacia@gmail.com; 6 Laboratory of Radiopharmacy and Nanoradiopharmaceuticals, Zona Oeste State University, Rio de Janeiro 23070-200, Brazil; 7 Department of Chemistry, Clemson University, Clemson, SC 29634, USA; dwhiteh@clemson.edu; 8 Department of Chemical Engineering, Polytechnique Montreal, Montreal, QC H3C 3A7, Canada

**Keywords:** calcium fluoride, particles, biocompatibility, ultrasound, X-ray, bioimaging, biomaterial

## Abstract

Ultrasound (US) and X-ray imaging are diagnostic methods that are commonly used to image internal body structures. Several organic and inorganic imaging contrast agents are commercially available. However, their synthesis and purification remain challenging, in addition to posing safety issues. Here, we report on the promise of widespread, safe, and easy-to-produce particulate calcium fluoride (*part*-CaF_2_) as a bimodal US and X-ray contrast agent. Pure and highly crystalline *part*-CaF_2_ is obtained using a cheap commercial product. Scanning electron microscopy (SEM) depicts the morphology of these particles, while energy-dispersive X-ray spectroscopy (EDS) confirms their chemical composition. Diffuse reflectance ultraviolet-visible spectroscopy highlights their insulating behavior. The X-ray diffraction (XRD) pattern reveals that *part*-CaF_2_ crystallizes in the face-centered cubic cell lattice. Further analyses regarding peak broadening are performed using the Scherrer and Williamson–Hall (W-H) methods, which pinpoint the small crystallite size and the presence of lattice strain. X-ray photoelectron spectroscopy (XPS) solely exhibits specific peaks related to CaF_2_, confirming the absence of any contamination. Additionally, in vitro cytotoxicity and in vivo maximum tolerated dose (MTD) tests prove the biocompatibility of *part*-CaF_2_. Finally, the results of the US and X-ray imaging tests strongly signal that *part*-CaF_2_ could be exploited in bimodal bioimaging applications. These findings may shed a new light on calcium fluoride and the opportunities it offers in biomedical engineering.

## 1. Introduction

Currently, ultrasound (US) echography (also known as sonography) and X-ray radiography are among the most frequently employed techniques in bioimaging [1,2,3]. Several materials have been designed to serve as contrast agents to improve the quality of the acquired images. In the case of X-ray imaging, iodine- and barium sulfate-based compounds are among the most widely used radiocontrast agents [4,5]. Similarly, molecules that absorb ultrasound irradiation have been designed using inert gas encapsulated into shell particles. The shell particles can be composed of albumin, lipids, galactose, or perflutren. On one hand, the main limitation of US bioimaging agents is associated with the lack of targeting and accumulation into tissues, which limits the imaging to the vascular space. On the other hand, the main limitation of X-ray bioimaging agents is associated with their short half-life and safety concerns. Therefore, it is challenging to prepare a bimodal X-ray and ultrasound bioimaging contrast agent with increased stability, an enhanced contrast signal, biocompatibility, and the ability to accumulate into tissues [6,7,8]. Such limitations might be overcome by the design of inorganic nanomaterials that are highly biocompatible and stable in biological fluids and are prone to the easy surface functionalization in order to improve their pharmacokinetic features while achieving tissue targeting [9,10]. To achieve this, in a previous article we described the synthesis of polymeric nanoparticles made of polytetrafluoroethylene-like material (PTFE ≈ NPs), as well as their physico-chemical characterization, biological properties, and their promise in the biomedical field as a contrast agent for bimodal ultrasound and X-ray bioimaging [11]. However, PTFE is a very stable and chemically inert polymer that could have impurities. While PTFE polymer could be useful for some applications, there is a need to develop inorganic and biodegradable dual contrast agents for X-ray and ultrasound imaging.

Calcium fluoride (CaF_2_), an inorganic compound also known as fluorite, exhibits excellent biocompatibility [12,13], biodegradability [14,15,16,17], antibacterial activity [18], and antibiofilm properties [19]. Therefore, this material holds tremendous promise for biomedical applications. For instance, it was tested for wound healing when formulated in hydrogels [20], ^19^F magnetic resonance imaging [21], photoluminescence biosensing when doped with Mn^2+^ [22], biomedical imaging when doped by Tb^3+^/Gd^3+^ [23], and for targeted labeling of cancer cells when doped with Eu^3+^ [12]. Due to its high fluorine content, most of the studies are limited to the potential of calcium fluoride in dental applications [16,24,25,26]. On the other hand, fluorinated compounds, such as octafluoropropane and perfluorobutane, that are formulated with proteins or lipids are already commercialized as ultrasound (US) contrast agents [27].

In the present article, we describe the preparation of CaF_2_ particles from a cheap commercial sealant product and their extensive physico-chemical characterization using several techniques. We also describe their excellent biocompatibility, studied both in vitro and in vivo, and highlight their outstanding features as a bimodal ultrasound and X-ray contrast agent for biomedical imaging applications.

## 2. Results and Discussion

### 2.1. Physicochemical Characterization

#### 2.1.1. Scanning Electron Microscopy Analysis

**Figure 1:**
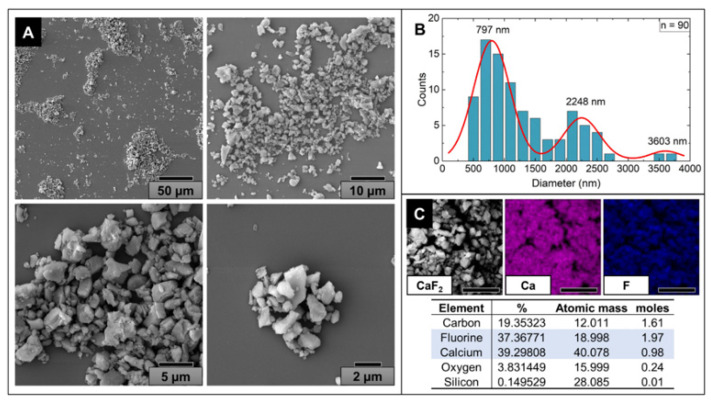
(**A**) SEM micrographs of *part*-CaF_2_ at different magnifications. (**B**) Particle size distribution of *part*-CaF_2_ (*n* = 90). (**C**) EDX elemental chemical analysis of *part*-CaF_2_: CaF_2_ (grey); Ca (red); and F (blue) (scale bar = 10 μm).

We describe a simple and cheap method to obtain high-purity, crystalline particulate calcium fluoride (*part*-CaF_2_) starting from a commercially available sealant for applications as a bimodal contrast agent in ultrasound and X-ray bioimaging. SEM analysis of the resulting white powder showed particles of irregular shapes and different sizes, where the size distribution ranged from a few hundred nanometers to a few microns (Figure 1A,B). However, the size of most of the particles spanned from 500 nm to 2 μm. Although the optimum size depends on the targeted bioapplications [9], the *part*-CaF_2_ generated in this study fell within the range of liposomes and polymeric particles used in drug delivery systems and biomedical imaging [9,10]. Moreover, various methods enabled the size control of the CaF_2_ particles to fit a specific application [16,20,24,28]. Additionally, EDX analysis showed the existence of various chemical elements at different concentrations, including calcium and fluorine (Figure 1C). However, the presence of carbon and oxygen may be ignored, as they most likely come from the adhesive layer used to prepare the sample for analysis. By dividing the molar percentages, a fluorine-to-calcium ratio of 2:1 was obtained, which fits the CaF_2_ chemical formula.

#### 2.1.2. X-ray Diffraction

**Figure 2:**
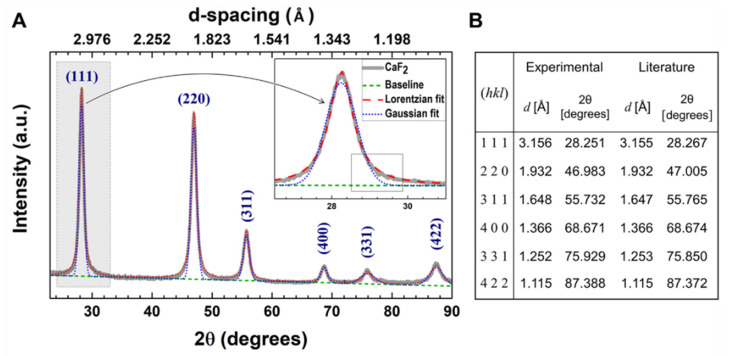
(**A**) Fitting of the *part*-CaF_2_ XRD pattern. (**B**) Peak assignment regarding positions and interplanar distances compared with the literature (JCPDS Card no 87-0971).

The XRD pattern of *part*-CaF_2_ (Figure 2A) displays typical peaks of pure CaF_2_ crystallizing in the face-centered cubic cell lattice (JCPDS Card no 87-0971) corresponding to the Fm3m crystalline group, in excellent agreement with published data (Figure 2B) [19,29]. Interestingly, the absence of extra peaks depicts the high purity of the obtained sample (cf. XPS). Prior to determining the cell lattice parameter, a Lorentzian function was applied for fitting purposes as it fits the XRD profile better when compared to the Gaussian analog (Insert Figure 2A). Using Equation (2), the lattice parameter, a, was determined to be 5.4615 Å, corroborating previously reported data [29,30,31]. Additionally, the index of crystallinity (IC) of *part*-CaF_2_ was estimated using Equation (3) to be 93.26%, denoting its high degree of crystallinity.

The determination of the crystallite size of *part*-CaF_2_ (Figure 2B) was carried out using low-angle and high-angle peaks and Equation (5). Nonetheless, low-range analysis provided more precise results because higher angles cause distortion and hence poor precision [32]. *part*-CaF_2_ exhibited a relatively small crystallite size of about 8–14 nm, values that were slightly smaller than those reported in published data [29,33]. Besides, the average crystallite size was significantly smaller than the particle size provided by SEM micrographs (Cf. Figure 1), demonstrating that the *part*-CaF_2_ sample was polycrystalline. Using ultrasonic probes or ball milling, it was possible to tune the particle size and downsize it to values approaching the crystallite size by breaking down the agglomerates bonded by van der Waals (vdW), capillary, or electrostatic interactions to give rise to a homogenous sample of much smaller particles that might be considered as monocrystalline [34,35,36].

#### 2.1.3. Diffuse Reflectance UV-Vis Spectroscopy

**Figure 3:**
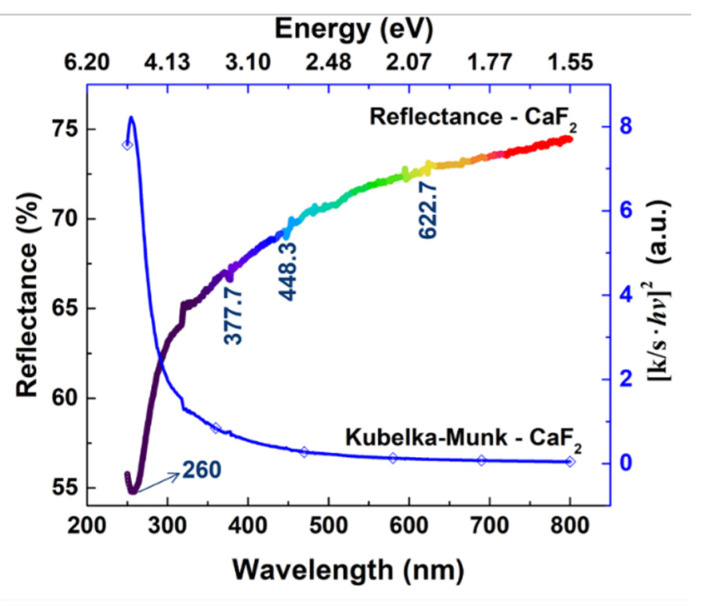
Diffuse reflectance UV-Vis spectrum of *part*-CaF_2_ (bottom and left axes) and its corresponding K-M transformation (upper and right axes).

The reflectance of *part*-CaF_2_ was recorded to study its optical properties (Figure 3). It clearly shows that the reflectance of *part*-CaF_2_ was ca. 55% in the lower limit of the spectrum at *λ* = 260 nm and increased steadily in the visible region without plateauing nor reaching its maximum value, as the recorded value at 800 nm was smaller than 75%. In other words, *part*-CaF_2_ partially absorbed the incident radiations in either the UV or visible regions. Moreover, the reflectance spectrum displayed weak peaks pointing down at 377.7 nm, 448.3 nm, and 622.7 nm, which may arise from the large surface-to-volume ratio exhibited by *part*-CaF_2_ [29]. Besides, the intense peak, observed at 260 nm, was attributed to surface defect absorption, such as Schottly and Frenkel, dangling bonds, and regions of disorder, which are common in particles [29,37]. On the other hand, the Kubelka–Munk (K-M) transformation of *part*-CaF_2_ did not permit the determination of the band gap since it is found in the high-ultraviolet region at around 10 eV [38]. However, *part*-CaF_2_ may be considered as a promising insulator, with a lattice constant similar to silicon due to its very large band gap [39].

#### 2.1.4. X-ray Photoelectron Spectroscopy

**Figure 4:**
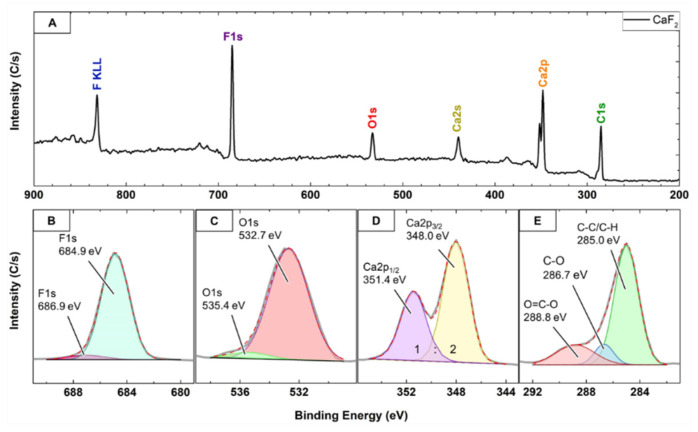
X-ray photoelectron spectra of *part*-CaF_2_. (**A**) Survey spectrum confirming the main characteristic atomic components. (**B**) F1s deconvolution. (**C**) O1s deconvolution. (**D**) Typical Ca2p spin-orbit splitting for Ca(II). (**E**) Chemical environment of the C1s peak.

Figure 4A displays the XPS general survey spectrum of *part*-CaF_2_ calibrated at C1s in C-C/C-H with a binding energy (BE) of 285.0 eV. It clearly exhibits two prominent peaks arising from the two elements constituting this sample, that is, fluorine (F1s) and calcium (Ca2p); it also exhibits less intense peaks related to carbon (C1s) and oxygen (O1s). The C1s peak can be deconvoluted into its 3 components, C-C/C-H, C-O, and C=O, centered at 285.0 eV, 285.7 eV, and 288.8 eV, respectively (Figure 4E) [40]. The C1s peak most likely originated from a background source, i.e., the tape used to mount the sample onto the XPS holder [30,36]. On the other hand, the deconvolution of the peak O1s revealed the presence of signals that correspond to O^2−^ (532.7 eV) and OH^−^ (535.4 eV) due to the well-known adsorption of H_2_O molecules on the CaF_2_ (111) plane (Figure 4C) [30,36]. This fact is also reflected in the high-resolution F1s peak (Figure 4B) that displays a very prominent peak centered at 684.9 eV, assigned to F-Ca bonding, in addition to a very weak peak centered at 686.9 eV that is due to F^−^ defects at the surface of CaF_2_, most likely originating from water adsorption [28,30,36]. Moreover, the Ca2p peak displayed a doublet owing to the spin-orbit splitting typical of Ca(II) assigned to Ca2p_1/2_ and Ca2p_3/2_, centered at 351.4 eV and 348.0 eV, respectively, and with 1:2 area ratio (Figure 4D) [28,30,36,40,41,42,43]. Finally, the XPS analysis enabled the estimation of the F:Ca atomic ratio to equal 2.04, highlighting the chemical formula of CaF_2_ and corroborating the SEM-EDX findings.

### 2.2. Biological Properties of Part-CaF_2_

#### 2.2.1. In Vitro Cytotoxicity

**Figure 5:**
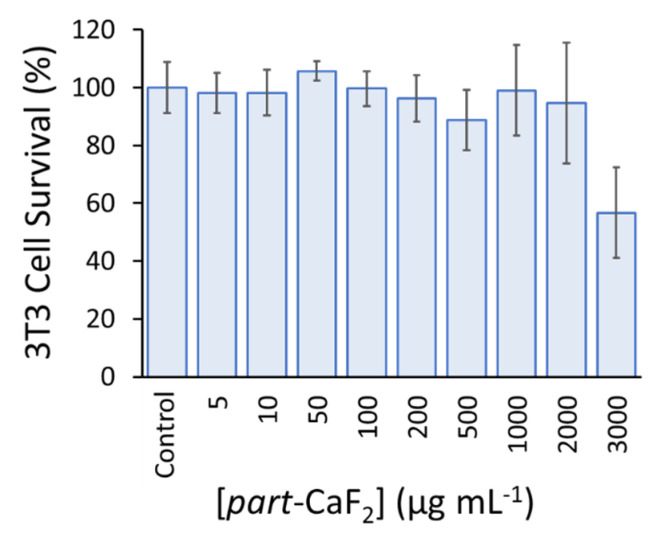
Cell viability of 3T3 fibroblast cell line incubated for 24 h with different concentrations of *part*-CaF_2_. The experiments were performed in 6 replicates.

The in vitro toxicity of various concentrations of *part*-CaF_2_ against 3T3 fibroblasts was studied via MTT assay (Figure 5). Compared to the control group, the 3T3 fibroblasts maintained their full viability (90–100%) for *part*-CaF_2_ concentrations of up to 2000 µg·mL^−1^ and 60% at the highest tested concentration (3000 µg·mL^−1^). These results highlight the excellent biocompatibility exhibited by these particles, making them suitable candidates for numerous applications in the biomedical field.

#### 2.2.2. Determination of Maximum Tolerated Dose

**Figure 6:**
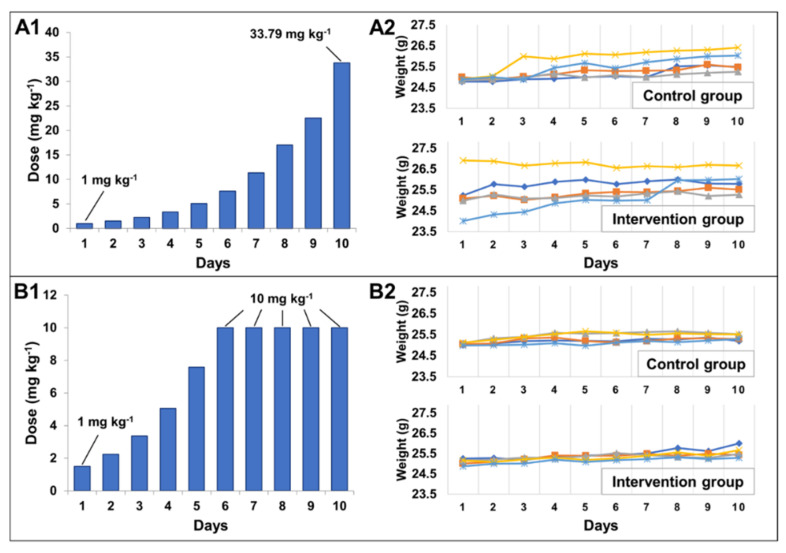
(**A1**) First MTD methodology for *part*-CaF_2_. The doses were incremented by no more than 50% from the initial dose over 10 days. (**B1**) Second MTD methodology for *part*-CaF_2_. The doses were doubled every day starting at 1.00 mg·kg^−1^. (**A2**,**B2**) For both methodologies, the weight loss was monitored for the control and intervention groups of 5 mice each.

In the first methodology, the initial dose for each animal started at 1.00 mg·kg^−1^ and all the doses were staggered up to 33.79 mg·kg^−1^ (Figure 6A1). In the second methodology (based on the ICH M3-R2), the initial dose for each animal started at 1 mg·kg^−1^, up to the maximum dose of 10.00 mg·kg^−1^ (Figure 6A2). The test was stopped on day 10 for both methodologies since the animals had not shown any notable variation in the parameters described in Table 1 (such as weight loss over 20%), or because none of the scores were over 2, as clearly depicted in Figure 6A2,B2. For both methodologies, the weight of all animals increased, and no clinical signs were observed (Figure 6). Therefore, it is possible to affirm that *part*-CaF_2_ is safe up to the dose of 33.79 mg·kg^−1^. Due to limitations in the amount of material produced, it was not possible to check the maximum tolerated dose (MTD) itself. However, even a very high dose shows no adverse effect on the animals.

### 2.3. Ultrasound and X-ray Imaging

**Figure 7:**
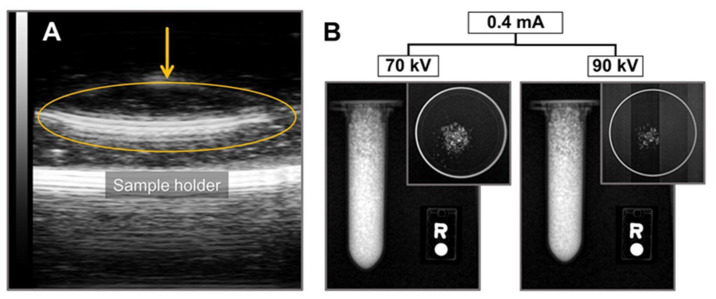
(**A**) Ultrasound imaging showing *part*-CaF_2_ suspended in water (orange arrow). (**B**) X-ray image showing a 2 mL Eppendorf tube filled with *part*-CaF_2_ and a top view of powder dispersed into a petri dish subjected to different potential and the same current intensity.

The ultrasound (US) imaging test, performed by subjecting an Eppendorf tube filled with *part*-CaF_2_ (Figure 7A) to US irradiation, demonstrated the US absorption properties of *part*-CaF_2_. In this image, *part*-CaF_2_ was suspended in water to reproduce a tissue environment (performed by casting the *part*-CaF_2_ into a flat petri dish), showing that US attenuation was exclusively generated by *part*-CaF_2_ reaching almost its maximum intensity. US attenuation occurs with sonosensitizers when the cavitation, followed by the collapse of small bubbles, produces enough energy to induce heating [6]. Although the mechanism of sonosensitizer excitation is not fully understood, most molecules exhibiting US attenuation contain halogen atoms in their structure [6], a common feature shared by commercially available US contrast agents [8], polytetrafluorethylene [11], and the *part*-CaF_2_ described in the present work. Thus, the results suggest that fluorine atoms from *part*-CaF_2_ likely contribute to the US absorption. However, the presence of halogen atoms in the molecular structure is not a requisite for US attenuation by a given material, as reported recently [44]. To the best of our knowledge, this is the first report showing the US imaging properties of *part*-CaF_2_ materials. These outstanding results may elicit interest in calcium fluoride for use in biomedical imaging applications.

Additionally, *part*-CaF_2_ exhibits X-ray attenuation, which is supported by the contrast signal observed in Figure 7B. Indeed, *part*-CaF_2_ neatly attenuates X-rays of different energies (i.e., different potentials applied at the same intensity). Moreover, the same material was subjected to different parameters by tuning the tube current and potential without affecting X-ray attenuation. This result highlights the capability of *part*-CaF_2_ to absorb the incident electromagnetic radiation of high energy X-rays at different conditions without affecting the yield.

As a contrast agent for both US and X-ray imaging, CaF_2_ may offer tremendous advantages when compared to analog molecules and particles either already used or in development for the same purpose [5,7,8]. In fact, this material is widespread in nature as a mineral. It is cheap and easy to produce via various methodologies [29,45,46,47], and may be water-soluble [48]. This should make its large-scale production easily achievable at very competitive costs. Besides, it has been already demonstrated that this biomaterial is amenable to entrapment within hydrogels [17,20], polymers [26], and resins [46,49]. All these facts presage an easy and versatile surface chemistry aiming at greater targeting efficiency and even increased biocompatibility, thus paving the way for other clinical uses in the biomedical field.

## 3. Materials and Methods

### 3.1. Preparation of Particulate CaF_2_

In this step, 0.3 g of a gray pipe thread compound (ACE^®^) and 2 mL of 40% aq. hydrofluoric acid (HF) were mixed in a Falcon tube and subjected to sonication for 2 h in an ultrasonic bath. As a result, 3 phases appeared. In addition to a dark gray substance (excipients) at the top and a translucent supernatant as the intermediate phase, the bottom phase consisted of a precipitate. This pellet, made of particulate CaF_2_ (*part*-CaF_2_), was collected and mixed with another 2 mL of 40% aq. HF in a Falcon tube and subjected to sonication for 45 min in an ultrasonic bath. The sample was then centrifuged at 2000 rpm for 5 min. Finally, the pellet of *part*-CaF_2_ was collected and washed 3 times using isopropyl alcohol, dried in an oven at 80 °C for 3 h, and stored for further analysis and investigation.

### 3.2. Physico-chemical Characterization

#### 3.2.1. Scanning Electron Microscopy—Energy Dispersive X-ray Spectroscopy (SEM-EDX)

The scanning electron microscopy analysis of the *part*-CaF_2_ sample was carried out using a TESCAN FEG SEM MIRA3 apparatus. *part*-CaF_2_ powder was fixed onto SEM stubs for analysis using a carbon adhesive layer and sputter-coated with an approximately 20 nm gold (99.99% purity) layer. The EDX analysis was performed using the same device since it is equipped with a Bruker X-Flash 6|30 detector, with 123 eV resolution at Mn Kα with an EDX detector.

#### 3.2.2. X-ray Diffraction

X-ray diffraction analysis was carried out on the *part*-CaF_2_ sample using a PANalytical brand *θ*–2*θ* configuration (Bragg–Brentano geometry) X-ray tube, with Cu Kα irradiation *λ* = 1.54059 Å in an EMPYREAN diffractometer. The acquired XRD pattern was fitted using OriginPro software to determine the lattice parameters, index of crystallinity, and crystallite size. The interplanar distance, *d*, was determined using Bragg’s law (Equation (1)).

(1) n λ=2 dsinθ

where *n* is the order of reflection (*n* = 1), and *λ* is the wavelength of Cu Kα irradiation. Then, the main peaks were compared with reliable databases to assign Miller indices, (hkl), where a face-centered cubic crystal system for CaF_2_ was determined for which the space group was Fm3m. Therefore, there were 3 equal axes at right angles, for which the lattice parameter value was computed using Equation (2):

(2) $$\frac{1}{d^{2}}=\frac{h^{2}+k^{2}+l^{2}}{a^{2}}$$

The lattice parameter *a* was calculated using the interplanar distance of the most intense peak (111). Besides, the index of crystallinity (IC) of the *part*-CaF_2_ sample was determined by comparing the ratios between the areas of crystalline peaks (crystalline phase) and of their respective background (amorphous phase) using the empirical Equation (3):

(3) $$\mathrm{IC}=\frac{\mathrm{Area}\;\mathrm{of}\;\mathrm{Crystalline}\;\mathrm{Peaks}}{\mathrm{Total}\;\mathrm{Area}\;\left(\mathrm{Crystalline}\;+\;\mathrm{Amorphous}\right)}\times 100\%$$

In addition, the crystallite size was determined by analyzing the breadth peak. Instrumental and physical broadening effects were considered as broadening of the Bragg peaks (Equation (4)) [50]:

(4) $$\beta_{hkl}={\left[{\left(\beta_{hkl}\right)}_{\mathrm{measured}}^{2}-{\left(\beta_{hkl}\right)}_{\mathrm{instrumental}}^{2}\right]}^{1/2}$$

The broadening due to the instrumental setup was corrected with a previous diffraction pattern using a silicon standard specimen. Then, substitution of Equation (4) into the well-known Scherrer equation yields the following formulation (Equation (5)):

(5) $$\beta_{hkl}={\left(\beta_{hkl}\right)}_{\mathrm{measured}}=K\lambda {\left(L\;\cos\left(\chi /2\right)\right)}^{-1}$$

where *β_hkl_* is the breadth value of the full width at half maximum (FWHM) taken on a 2*θ* scale (transformed into radians); *K* is a numerical constant equal to 0.94; *θ* is the Bragg angle taken on a 2*θ* scale (in degrees); *λ* = 1.54059 Å; and *L* is the linear dimension of the particle or crystallite size. The crystallite size is the average of values calculated for each diffraction peak.

Further details regarding the XRD analysis of *part*-CaF_2_ powder are provided in the Supporting Information (SI).

#### 3.2.3. Diffuse Reflectance UV-Vis Spectroscopy

Diffuse Reflectance UV-Vis spectroscopy was performed on *part*-CaF_2_ powder to determine its optical characteristics. The “LAMBDA 1050 UV-Vis Spectrophotometer PerkinElmer^®^, equipped with a PerkinElmer^®^ accessory 3D WB Detector Module and the Praying Mantis™ Diffuse Reflection Accessory, was employed. The light spot was about 1–2 mm. The powder was placed in the sample holder (a hole of 10 mm in diameter and 3 mm depth) and the surface was flattened. A white standard of BaSO_4_ was used as blank prior to measurement. Band gap analysis was carried out using the Kubelka–Munk (K-M) function [11]. The x-axis (wavelength) was converted to energy, E, by applying the Einstein–Planck relation (Equation (6)):

(6) $$E=h\nu =h\;\frac{c}{\lambda }$$

where *h* is the Planck constant (4.135667 × 10^−15^ eV), c is the speed of light, and *λ* is the wavelength. The y-axis (reflectance) was converted to [k/s⋅hν]^2^ by applying the Kubelka–Munk function (Equation (7)):

(7) $$\frac{k}{s}=\frac{{\left(1-R\right)}^{2}}{2R}$$

where *k* is the absorption coefficient, *s* is the scattering coefficient, and *R* is the reflectance. The value of the band gap was determined graphically by extrapolating a straight line at *k* = 0.

#### 3.2.4. X-ray Photoelectron Spectroscopy (XPS)

The XPS spectrum of the *part*-CaF_2_ sample was recorded using a Thermo VG ESCALAB 250 (East Grinstead, UK) fitted with a monochromated Al Kα X-ray source with an incident energy of 1486.6 eV. An electron flood gun was used for charge compensation. The analyzer was operated at 40 and 100 eV pass energy for the narrow regions and survey spectra, respectively. Elemental atomic concentrations were calculated from the XPS peak areas and the corresponding Scofield sensitivity factors corrected for the analyzer transmission work function. The measurement was conducted at 255 eV survey operation pass energy and 55 eV for high-resolution narrow regions. Prior to the analysis, the recorded spectrum was calibrated with C1s peak in C-C/C-H at 285.0 eV, followed by fitting and deconvolution of the relevant XPS peaks using the Gauss–Lorentzian function [40,43].

### 3.3. Biological Properties of Part-CaF_2_

#### 3.3.1. In Vitro Cytotoxicity

This was carried out using a routine assay that is commonly used to study the toxicity of nanomaterials. Namely, 3T3 fibroblast cells were seeded in 96-well plates at a concentration of 104 cells per well in 100 μL of DMEM medium containing 10% FBS and 1% penicillin–streptomycin. The cells were then incubated overnight at 37 °C under a controlled atmosphere (5% CO_2_ and 80% H_2_O). Next, the culture medium was replaced by the same medium (100 µL) but containing variable concentrations of the *part*-CaF_2_ sample (0–3000 µg·mL^−1^). After 48 h of incubation, the wells were filled with 20 µL cell culture medium (MTS), and then incubated for another 4 h at 37 °C. UV absorbance was measured at 490 nm with a microplate reader (Varioskan Flash, Thermo Scientific, Waltham, MA, USA). Experiments were carried out in 6 replicates and expressed as a percentage of viable cells compared to the control group.

#### 3.3.2. Maximum Tolerated Dose (MTD)

All animals were obtained from the Federal University of Rio de Janeiro Facility and housed under specific pathogen-free conditions. Healthy male Swiss mice (*n* = 20) aged between 8 and 10 weeks were kept in cages (3 per cage) at a controlled temperature (24–25 °C), and received food and water ad libitum. All experiments were conducted according to the IPEN Animal Ethics Committee approvals (Protocols IPEN-182/2018) and Guidance on Nonclinical Safety Studies for the Conduct of Human Clinical Trials and Market Authorization for Pharmaceuticals (ICH-M3(R2)/2009).

The maximum tolerated dose (MTD) of *part*-CaF_2_ that did not induce unacceptable side effects or toxicity over a specific period of time was determined by 2 methodologies based on both weight loss and clinical signs [51]. For the first methodology, 10 mice were dosed daily. The initial dose for each animal started at 1 mg·kg^−1^ and all the doses were staggered until reaching 33.79 mg·kg^−1^, following the system: day 1 dose = 1 mg·kg^−1^; day 2 dose = day 1 dose + 50%, and so on until day 10, with a final dose of 33.79 mg·kg^−1^. For the second methodology, the other 10 mice were dosed based on the ICH M3-R2. The initial dose for each animal started at 1 mg·kg^−1^ until the maximum dose of 10 mg·kg^−1^ was reached on day 6. In the last 4 days, all the mice received a fixed dose of 10 mg·kg^−1^. The clinical signs were scored based on observation of: (i) general appearance and (ii) body condition. The score was based on the following punctuation: 0 = normal; 1 = slight deviation from normal; and 2 = moderate deviation from normal, as established in Table 1.

### 3.4. Ultrasound and X-ray Imaging Using Part-CaF_2_

The absorption of ultrasounds (US) by the *part*-CaF_2_ sample was determined at the Mouse Clinical Institute (Clemson University, Clemson, SC, USA) using a preclinical Vevo 2100 echographie and computer (Visualsonics, Toronto, ON, Canada). The parameters employed were as follows—frequency: 21 MHz; power: 100%; acquisition depth and width of 16.00 mm and 23.04 mm, respectively.

The X-ray attenuation by *part*-CaF_2_ powder was evaluated in Eppendorf tubes and Petri dishes using a small preclinical micro-CT scanner (1076 Skyscan, Kartuizersweg, Belgium). The experimental parameters were set as follows: X-ray: 70 kV, 90 kV, 0.4 mA, 1 mA; resolution: 35 mm; pitch: 0.4; aluminum filters: 0.5 and 632 ms.

## 4. Conclusions and Perspectives

Here, we described the facile production of calcium fluoride particles that are highly crystalline and pure according to XRD and XPS analyses. Compared to other bimodal imaging particles, our results support that *part*-CaF_2_ could offer some tremendous advantages, such as ease of synthesis, low cost, and biodegradability. UV-Vis analysis revealed that *part*-CaF_2_ possesses no absorbance in the visible region. Additionally, XPS confirmed the chemical composition and formula of the material, while the XRD pattern determined its characteristics in terms of the lattice system, cell parameters, and index of crystallinity. Importantly, both in vitro and in vivo experiments using *part*-CaF_2_ did not show any toxicity. Lastly, X-ray and US attenuation tests demonstrated that *part*-CaF_2_ could be used as a bimodal bioimaging contrast agent. Overall, our findings demonstrated that the *part*-CaF_2_ sample holds a great promise for biomedical imaging and opens future potential clinical bioimaging applications. Future work will aim at controlling the size of the particles while maintaining the bimodal contrast signal, implementing the right surface coating, and translating our findings into animal models to understand the biodistribution and pharmacokinetics of this biomaterial and assess its dual bioimaging efficiency in living animals.

## Figures and Tables

**Table 1:** Dose escalation decision of MTD for clinical observations.

Score		0	1	2
Appearance	Weight loss	Steady weight	>10% weight loss	15–20% weight loss
	Coat	Normal	Mild ruffled coat	Moderate ruffled coat
	Body condition	Normal	Thin	Excessive loss fat (>15%)
	Body posture	Normal	Hunched	Hunched and still
	Movement	Normal	Reduced/slow	Reluctant to move when touched
Activity	Proximity to others	Close contact	Separate	Completely separate
Other	Injection site	Normal	Redness	Redness and swelling

## Data Availability

Data are contained within the article.

## Supplementary Materials

The following are available, X-ray Diffraction Analysis; Figure S1. W-H analysis of part-CaF2. Crystallite size was computed from the intercept value, whereas energy density, strain and stress were deduced from the slope, Figure S2. SSP plot for crystallite size analysis of part-CaF2, Figure S3. Logarithm-Scherrer plot for crystallite size analysis of part-CaF2, Table S1. Geometric parameters, including crystallite size for part-CaF2 powder using different methods. Low-range consideration points out the analysis of peaks at lower angles (111), (220) and (311); whereas whole-range alludes to all peak analysis.

## Sample Availability

Samples of the compounds are not available from the authors.

## References

[1] Nelson T.R., Pretorius D.H. (1998). Three-Dimensional Ultrasound Imaging. Ultrasound Med. Biol..

[2] Bercovich E., Javitt M.C. (2018). Medical Imaging: From Roentgen to the Digital Revolution, and Beyond. Rambam Maimonides Med. J..

[3] Wallyn J., Anton N., Akram S., Vandamme T.F. (2019). Biomedical Imaging: Principles, Technologies, Clinical Aspects, Contrast Agents, Limitations and Future Trends in Nanomedicines. Pharm. Res..

[4] Zhou S.A., Brahme A. (2008). Development of phase-contrast X-ray imaging techniques and potential medical applications. Phys. Med..

[5] Lusic H., Grinstaff M.W. (2013). X-ray-computed tomography contrast agents. Chem. Rev..

[6] Shibaguchi H., Tsuru H., Kuroki M., Kuroki M. (2011). Sonodynamic Cancer Therapy: A Non-invasive and Repeatable Approach Using Low-intensity Ultrasound with a Sonosensitizer. Anticancer Res..

[7] Chen H., Zhou X., Gao Y., Zheng B., Tang F., Huang J. (2014). Recent progress in development of new sonosensitizers for sonodynamic cancer therapy. Drug Discov. Today.

[8] Ignee A., Atkinson N.S., Schuessler G., Dietrich C.F. (2016). Ultrasound contrast agents. Endosc. Ultrasound.

[9] Alexis F., Pridgen E., Molnar L.K., Farokhzad O.C. (2008). Factors Affecting the Clearance and Biodistribution of Polymeric Nanoparticles. Mol. Pharm..

[10] Kolishetti N., Alexis F., Pridgen E.M., Farokhzad O.C. (2011). Biodistribution and Pharmacokinetics of Nanoprobes. Nanoplatform-Based Molecular Imaging.

[11] Chingo Aimacaña C.M., Quinchiguango Perez D.A., Rocha Pinto S., Debut A., Attia M.F., Santos-Oliveira R., Whitehead D.C., Terencio T., Alexis F., Dahoumane S.A. (2021). Polytetrafluoroethylene-like Nanoparticles as a Promising Contrast Agent for Dual Modal Ultrasound and X-ray Bioimaging. ACS Biomater. Sci. Eng..

[12] Sasidharan S., Jayasree A., Fazal S., Koyakutty M., Nair S.V., Menon D. (2013). Ambient temperature synthesis of citrate stabilized and biofunctionalized, fluorescent calcium fluoride nanocrystals for targeted labeling of cancer cells. Biomater. Sci..

[13] Prasad S., Ganisetti S., Jana A., Kant S., Sinha P.K., Tripathy S., Illath K., Ajithkumar T.G., Annapurna K., Allu A.R. (2020). Elucidating the effect of CaF_2_ on structure, biocompatibility and antibacterial properties of S53P4 glass. J. Alloys Compd..

[14] Afseth J., Ekstrand J., Hagelid P. (1987). Dissolution of calcium fluoride tablets in vitro and bioavailability in man. Scand. J. Dent. Res..

[15] Larsen M.J., Ravnholt G. (1994). Dissolution of Various Calcium Fluoride Preparations in Inorganic Solutions and in Stimulated Human Saliva. Caries Res..

[16] Sun L., Chow L.C. (2008). Preparation and properties of nano-sized calcium fluoride for dental applications. Dent. Mater..

[17] Ghafar H., Khan M.I., Sarwar H.S., Yaqoob S., Hussain S.Z., Tariq I., Madni A.U., Shahnaz G., Sohail M.F. (2020). Development and Characterization of Bioadhesive Film Embedded with Lignocaine and Calcium Fluoride Nanoparticles. AAPS PharmSciTech.

[18] Bala W.A., Benitha V.S., Jeyasubramanian K., Hikku G.S., Sankar P., Kumar S.V. (2017). Investigation of anti-bacterial activity and cytotoxicity of calcium fluoride nanoparticles. J. Fluor. Chem..

[19] Kulshrestha S., Khan S., Hasan S., Khan M.E., Misba L., Khan A.U. (2016). Calcium fluoride nanoparticles induced suppression of *Streptococcus mutans* biofilm: An in vitro and in vivo approach. Appl. Microbiol. Biotechnol..

[20] Jeong S.H., Shin D.Y., Kang I.K., Song E.H., Seong Y.J., Park J.U., Kim H.E. (2018). Effective Wound Healing by Antibacterial and Bioactive Calcium-Fluoride-Containing Composite Hydrogel Dressings Prepared Using in Situ Precipitation. ACS Biomater. Sci. Eng..

[21] Ashur I., Allouche-Arnon H., Bar-Shir A. (2018). Calcium Fluoride Nanocrystals: Tracers for In Vivo ^19^F Magnetic Resonance Imaging. Angew. Chem. Int. Ed. Engl..

[22] Wei J., Zheng W., Shang X., Li R., Huang P., Liu Y., Gong Z., Zhou S., Chen Z., Chen X. (2019). Mn^2+^-activated calcium fluoride nanoprobes for time-resolved photoluminescence biosensing. Sci. China Mater..

[23] Straßer M., Schrauth J.H.X., Dembski S., Haddad D., Ahrens B., Schweizer S., Christ B., Cubukova A., Metzger M., Walles H. (2017). Calcium fluoride based multifunctional nanoparticles for multimodal imaging. Beilstein J. Nanotechnol..

[24] Xu H.H., Moreau J.L., Sun L., Chow L.C. (2010). Novel CaF_2_ nanocomposite with high strength and fluoride ion release. J. Dent. Res..

[25] Azami M., Jalilifiroozinezhad S., Mozafari M., Rabiee M. (2011). Synthesis and solubility of calcium fluoride/hydroxy-fluorapatite nanocrystals for dental applications. Ceram. Int..

[26] Mitwalli H., Balhaddad A.A., AlSahafi R., Oates T.W., Melo M.A.S., Xu H.H.K., Weir M.D. (2020). Novel CaF_2_ Nanocomposites with Antibacterial Function and Fluoride and Calcium Ion Release to Inhibit Oral Biofilm and Protect Teeth. J. Funct. Biomater..

[27] Klibanov A.L. (1999). Targeted delivery of gas-filled microspheres, contrast agents for ultrasound imaging. Adv. Drug Deliv. Rev..

[28] Chandra Sekhar Reddy K., Chingakham C., Gupta B., Shiva Prasad M., Atchuta S.R., Sakthivel S. (2019). Single compound in-situ synthesis of core-shell CaF_2_ nanoparticles based broad band antireflective coatings for solar energy conversion. Sol. Energy.

[29] Pandurangappa C., Lakshminarasappa B.N. (2011). Optical absorption and Photoluminescence studies in Gamma-irradiated nanocrystalline CaF_2_. J. Nanomed. Nanotechnol..

[30] Bezerra C.d.S., Valerio M.E.G. (2016). Structural and optical study of CaF_2_ nanoparticles produced by a microwave-assisted hydrothermal method. Phys. B Condens. Matter.

[31] Il’ves V.G., Sokovnin S.Y., Zuev M.G., Uimin M.A., Rähn M., Kozlova J., Sammelselg V. (2019). Effect of Annealing on Structural, Textural, Thermal, Magnetic, and Luminescence Properties of Calcium Fluoride Nanoparticles. Phys. Solid State.

[32] Motevalizadeh L., Heidary Z., Ebrahimizadeh Abrishami M. (2014). Facile template-free hydrothermal synthesis and microstrain measurement of ZnO nanorods. Bull. Mater. Sci..

[33] Pandurangappa C., Lakshminarasappa B.N. (2011). Optical studies of samarium-doped fluoride nanoparticles. Philos. Mag..

[34] Taurozzi J.S., Hackley V.A., Wiesner M.R. (2011). Ultrasonic dispersion of nanoparticles for environmental, health and safety assessment—Issues and recommendations. Nanotoxicology.

[35] Robertson J.D., Rizzello L., Avila-Olias M., Gaitzsch J., Contini C., Magon M.S., Renshaw S.A., Battaglia G. (2016). Purification of Nanoparticles by Size and Shape. Sci. Rep..

[36] Molaiyan P., Witter R. (2019). Surface defect-enhanced conductivity of calcium fluoride for electrochemical applications. Mater. Des. Process. Comm.

[37] Kumar G.A., Chen C.W., Ballato J., Riman R.E. (2007). Optical Characterization of Infrared Emitting Rare-Earth-Doped Fluoride Nanocrystals and Their Transparent Nanocomposites. Chem. Mater..

[38] Heaton R.A., Lin C.C. (1980). Electronic energy-band structure of the calcium fluoride crystal. Phys. Rev. B.

[39] Suturin S.M., Banshchikov A.G., Sokolov N.S., Tyaginov S.E., Vexler M.I. (2009). Static current-voltage characteristics of Au/CaF_2_/n-Si(111) MIS tunneling structures. Semiconductors.

[40] Budyanto S., Kuo Y.-L., Liu J.C. (2015). Adsorption and precipitation of fluoride on calcite nanoparticles: A spectroscopic study. Sep. Purif. Technol..

[41] Fujii E., Kawabata K., Yoshimatsu H., Hayakawa S., Tsuru K., Osaka A. (2003). Structure and Biomineralization of Calcium Silicate Glasses Containing Fluoride Ions. J. Ceram. Soc. Jpn..

[42] Gerth H.U., Dammaschke T., Schafer E., Zuchner H. (2007). A three layer structure model of fluoridated enamel containing CaF_2_, Ca(OH)_2_ and FAp. Dent. Mater..

[43] Borisyuk P.V., Vasilyev O.S., Krasavin A.V., Lebedinskii Y.Y., Troyan V.I., Tkalya E.V. (2015). Band structure and decay channels of thorium-229 low-lying isomeric state for ensemble of thorium atoms adsorbed on calcium fluoride. Phys. Status Solidi C.

[44] Ono K. (2020). A Comprehensive Report on Ultrasonic Attenuation of Engineering Materials, Including Metals, Ceramics, Polymers, Fiber-Reinforced Composites, Wood, and Rocks. Appl. Sci..

[45] Yang Z., Wang G., Guo Y., Kang F., Huang Y., Bo D. (2012). Microwave-assisted synthesis and characterization of hierarchically structured calcium fluoride. Mater. Res. Bull..

[46] Łukomska-Szymańska M., Zarzycka B., Grzegorczyk J., Sokołowski K., Półtorak K., Sokołowski J., Łapińska B. (2016). Antibacterial Properties of Calcium Fluoride-Based Composite Materials: In Vitro Study. BioMed Res. Int..

[47] Malviya D., Pawade V.B., Bhanvase B.A. (2019). Ultrasound assisted synthesis of CaF_2_:Eu^3+^ phosphor nanoparticles. Luminescence.

[48] Wang J., Miao W., Li Y., Yao H., Li Z. (2009). Water-soluble Ln^3+^-doped calcium fluoride nanocrystals: Controlled synthesis and luminescence properties. Mater. Lett..

[49] Xu H.H., Moreau J.L., Sun L., Chow L.C. (2008). Strength and fluoride release characteristics of a calcium fluoride based dental nanocomposite. Biomaterials.

[50] Nath D., Singh F., Das R. (2020). X-ray diffraction analysis by Williamson-Hall, Halder-Wagner and size-strain plot methods of CdSe nanoparticles-a comparative study. Mater. Chem. Phys..

[51] Aston W.J., Hope D.E., Nowak A.K., Robinson B.W., Lake R.A., Lesterhuis W.J. (2017). A systematic investigation of the maximum tolerated dose of cytotoxic chemotherapy with and without supportive care in mice. BMC Cancer.

