# Supplementary material for: Bimodal Ultrasound and X-ray Bioimaging Properties of Particulate Calcium Fluoride Biomaterial
Source: Molecules. 2021 Sep 7;26(18):5447. doi: 10.3390/molecules26185447 (PMC8472579; doi:10.3390/molecules26185447)
Supplement: Supplementary file 1 [file molecules-26-05447-s001.zip › molecules-1341068-supplementary.pdf]

Supporting Information

Bimodal Ultrasound and X-ray Bioimaging Properties of Particulate Calcium Fluoride Biomaterial

Cristhian Marcelo Chingo Aimacaña [1], Kevin O. Pila [2], Dilan A. Quinchiguango Perez[2], Alexis Debut [3], Mohamed F. Attia [4], Ralph Santos-Oliveira [5,6], Daniel C. Whitehead [7], Carlos Reinoso [1], Frank Alexis [2*], Si Amar Dahoumane [2,8*]

[1] School of Physical Sciences and Nanotechnology, Yachay Tech University, 100650-Urcuquí, Ecuador; cristhian.chingo@yachaytech.edu.ec, creinoso@yachaytech.edu.ec
[2] School of Biological Sciences and Engineering, Yachay Tech University, 100650-Urcuquí, Ecuador; kevin.pila@yachaytech.edu.ec ; dilan.quinchiguango@yachaytech.edu.ec; falexis@yachaytech.edu.ec; sa.dahoumane@gmail.com
[3] Center of Nanoscience and Nanotechnology, Universidad de las Fuerzas Armadas ESPE, Sangolquí 170501, Ecuador; apdebut@espe.edu.ec
[4] Center for Nanotechnology in Drug Delivery and Division of Pharmaco-engineering and Molecular Pharmaceutics, Eshelman School of Pharmacy, University of North Carolina at Chapel Hill, Chapel Hill, NC 27599, USA; mattia@email.unc.edu
[5] Laboratory of Nanoradiopharmacy and Synthesis of Novel Radiopharmaceuticals, Nuclear Engineering Institute, Brazilian Nuclear Energy Commission, 21941906-Rio de Janeiro, Brazil.
[6] Laboratory of Radiopharmacy and Nanoradiopharmaceuticals, Zona Oeste State University, 23070200, Rio de Janeiro, Brazil; presidenciaradiofarmacia@gmail.com
[7] Department of Chemistry, Clemson University, Clemson, South Carolina 29634, USA, dwhiteh@clemson.edu
[8] Department of Chemical Engineering, Polytech Montreal, Montreal, H3C 3A7, Canada.
* Correspondence: falexis@yachaytech.edu.ec (FA); si-amar.dahoumane@polymtl.ca (SAD)

X-Ray Diffraction Analysis

The broadening due to the instrumental set-up was corrected in the previous diffraction pattern (cf. X-ray Diffraction analysis) with a silicon standard specimen. Then, the substitution of Eq. 4 into the well-known Scherrer's Equation yields Eq. S1:

$$\beta_{hkl} = (\beta_{hkl})_{measured} = K\lambda(L\,cos(\chi/2))^{-1} \quad \text{(Eq. S1)}$$

where β_{hkl} is the breadth value of the full width at half maximum (FWHM) taken on a 2θ scale (transformed into radians), K is a numerical constant equal to $2(ln\,2/\pi)^{1/2} = 0.93$; χ is the Bragg angle taken on a 2θ scale (in degrees); λ = 1.54059 Å; and L is called the linear dimension of the particle or crystallite size. The crystallite size is calculated for each diffraction peak and the average value provides the average crystallite size.

Including a logarithmic adjustment into Scherrer's Eq. S1 gives more precise results:

$$ln(\beta_{hkl}) = ln(K\lambda/L) + ln(cos(\chi/2)^{-1}) \quad \text{(Eq. S2)}$$

Then, plotting $ln(cos(\chi/2)^{-1})$ vs. $ln(\beta_{hkl})$ aids to obtain a linear fitting whose intercept (a) gives the crystallite size (L):

$$a = ln(K\lambda/L) \quad \text{(Eq. S3)}$$

The following methods consider the peak broadening of XRD as a consequence of the intrinsic strain effect in addition to the crystallite size. Such methods are in essence a modification of the well-known Williamson-Hall (W-H) method that is widely used to determine accurately the strain and crystallite size. W-H method claims that the total peak broadening is a product of both crystallite size and microstrain [1]. Uniform deformation model (UDM), uniform stress deformation model (USDM) and uniform deformation energy density model (UDEDM) modify the W-H equation to accurately determine the crystallite size. These cases are treated in order with the following basic relationship [1]:

$$\beta_{FWHM} = \beta_{hkl} + \beta_{strain} \quad \text{(Eq. S4)}$$

First, UDM considers crystal imperfections as a source of unidirectional strain across nanocrystals (isotropic nature of crystals). This strain takes place in the peak broadening as follows:[2]

$$\beta_{strain} = 4\varepsilon \cdot tan(\chi/2) \quad \text{(Eq. S5)}$$

The substitution of Eq. S1 and S5 into Eq. S4 yields Eq. S6:

$$\beta_{FWHM} = K\lambda(L\,cos(\chi/2))^{-1} + 4\varepsilon \cdot tan(\chi/2) \quad \text{(Eq. S6)}$$

where β_{FWHM} is the full width at half maximum value for individual peaks. Eq. S6 is rearranged as:

$$\beta_{FWHM} \cdot cos(\chi/2) = K\lambda(L)^{-1} + 4\varepsilon \cdot sin(\chi/2) \quad \text{(Eq. S7)}$$

Eq. S7, known as the UDM formulation, seems to be the straight-line equation. Plotting $4 \cdot sin(\chi/2)$ vs. $\beta_{FWHM} \cdot cos\,(\chi/2)$ aids to obtain a linear fitting, where the slope (b) provides the intrinsic strain (ε), while the intercept (a) gives the crystallite size.

The USDM confronts the assumption of the homogeneous and isotropic nature of crystals stated by the UDM. Instead, it modifies the W-H equation to assume the anisotropic nature of real crystals by including an anisotropic strain. It states a lattice deformation due to stress effects dispersed across all lattice planes containing micro-strain. Hooke's law establishes a relationship in the following way [3]:

$$\varepsilon = \sigma / Y_{hkl} \qquad \text{(Eq. S8)}$$

where σ is the stress and Y_{hkl} is the Young's modulus. Then, the substitution of Eq. S8 into Eq. S7 leads the following relation:

$$\beta_{FWHM} \cdot cos(\chi/2) = K\lambda(L)^{-1} + \frac{4\sigma}{Y_{hkl}} \cdot sin(\chi/2) \qquad \text{(Eq. S9)}$$

In Eq. S9, the anisotropic nature of Young's modulus is considered; it means a modified W-H equation with real crystal approximation. The Young's modulus for cubic structures is obtained for each diffraction peak by following:

$$\frac{1}{Y_{hkl}} = S_{11} - 2\left[(S_{11} - S_{12}) - \frac{1}{2} \cdot S_{44}\right]\left[\frac{h^2k^2 + k^2l^2 + l^2h^2}{\left(h^2 + k^2 + l^2\right)^2}\right] \qquad \text{(Eq. S10)}$$

where S_{11}, S_{12} and S_{44} known as elastic compliances were calculated from the elastic stiffness constants for CaF_2 by applying the following expressions:

$$S_{11} = \frac{C_{11} + C_{12}}{(C_{11} - C_{12}) \cdot (C_{11} + C_{12})} \qquad \text{(Eq. S11)}$$

$$S_{12} = \frac{-C_{12}}{(C_{11} - C_{12}) \cdot (C_{11} + 2C_{12})} \qquad \text{(Eq. S12)}$$

$$S_{44} = \frac{1}{C_{44}} \qquad \text{(Eq. S13)}$$

The stiffness values for C_{11}, C_{12} and C_{44} are 164.6 GPa, 44.4 GPa and 33.8 GPa, respectively [4]. Then the following plot of $4 \cdot sin(\chi/2)/Y_{hkl}$ vs. $\beta_{FWHM} \cdot cos(\chi/2)$ is made, whose slope will provide the stress (σ) whereas the intercept gives the average size (L). The intrinsic strain for the USDM method is obtained by applying Eq. S9.

The UDEDM method differs with the isotropic nature of crystals and the linear relation between strain and stress established by the UDM and USDM methods, respectively. Instead, it introduces agglomerations and dislocations as developers of imperfections in small crystals. Therefore, the Hooke's law of energy density is applied to justify the anisotropic lattice strain as a product of the deformation energy density (u).

$$\frac{\sigma}{Y_{hkl}} = \sqrt{\frac{2u}{Y_{hkl}}} \qquad \text{(Eq. S14)}$$

The combination of Eq. S14 and S9 yields Eq. S15:

$$\beta_{FWHM} \cdot cos(\chi/2) = K\lambda(L)^{-1} + 4 \cdot \sqrt{u} \cdot \sqrt{2/Y_{hkl}} \cdot sin(\chi/2) \qquad \text{(Eq. S15)}$$

Plotting $4 \cdot \sqrt{2/Y_{hkl}} \cdot sin(\chi/2)$ vs. $\beta_{FWHM} \cdot cos(\chi/2)$ aids to obtain the crystallite size (L) from the interception (a) and the energy density (u) from the slope (b). Eq. S8 and S14 are applied to calculate strain (ε) and stress (σ) [5].

Size strain plot (SSP) method similarly to W-H, takes into consideration peak broadening as a consequence of lattice strain. Notwithstanding the isotropic nature of crystals is considered, SSP gives excellent accurate results for size and lattice strain and is given by:

$$[d_{hkl} \cdot \beta_{FWHM} \cdot cos(\chi/2)]^2 = K\lambda(L)^{-1} \cdot d_{hkl}^2 \cdot \beta_{FWHM} \cdot cos(\chi/2) + \frac{\varepsilon^2}{4} \quad \text{(Eq. S16)}$$

where d_{hkl} is the interplanar distance for different peaks as shown in Eq. 2 for cubic crystals. Then, plotting $d_{hkl}^2 \cdot \beta_{FWHM} \cdot cos(\chi/2)$ vs. $[d_{hkl} \cdot \beta_{FWHM} \cdot cos(\chi/2)]^2$ is necessary, whose slope (b) gives the crystallite size (L), while the interception (a) provides the strain (ε) [1, 5].

W-H methods, logarithm-Scherrer and SSP are plotted in Figures S1, S2 and S3, respectively.

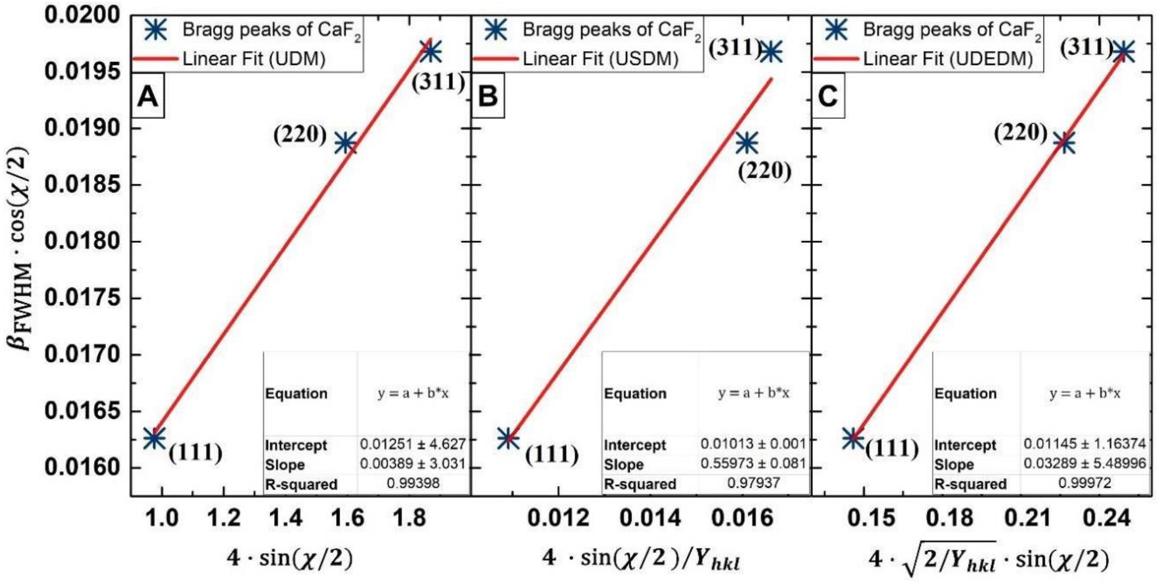

Figure S1. W-H analysis of *part*-CaF_2. Crystallite size was computed from the intercept value, whereas energy density, strain and stress were deduced from the slope.

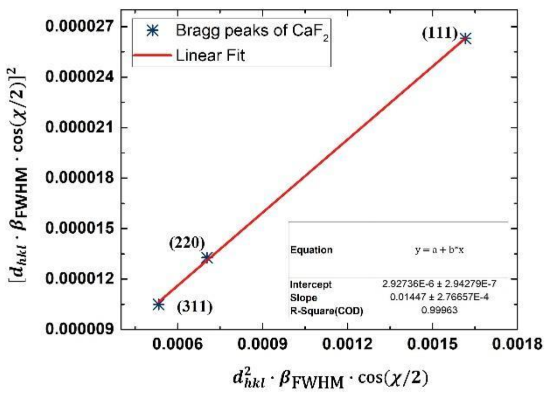

Figure S2. SSP plot for crystallite size analysis of *part*-CaF_2.

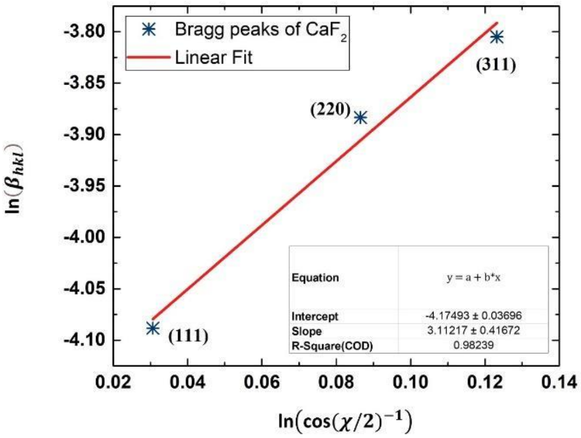

Figure S3. Logarithm-Scherrer plot for crystallite size analysis of *part*-CaF_2.

The crystallite size estimation (Table S1) was carried out using low-angle and high-angle peaks. However, low-range analysis provided more precise results because higher angles cause distortion and hence poorly precision [6]. Calcium fluoride shows a relatively small crystallite size of about 7.9-14.1 nm as shown in Table S1. The average crystallite size can be undoubtedly smaller or equal to sizes measured by other recognized techniques (TEM, small angle X-ray scattering, SEM).[2] However, analysis of XRD pattern is still considered as a reliable method even for grain size determination [3]. It is possible to approach the crystallite size as particle size with further methods such as ultrasonic probe, ball milling or differential centrifugation, in order to break up agglomerates bonded either by van der Waals (vdW), capillary or electrostatic interactions to pick from there the smallest particles [7-9].

Table S1. Geometric parameters, including crystallite size for *part*-CaF_2 powder using different methods. Low-range consideration points out the analysis of peaks at lower angles (*111*), (*220*) and (*311*); whereas whole-range alludes to all peak analysis.

Attention	Scherrer	Logarithm Scherrer	Williamson-Hall Method									Size Strain Plot (SSP)	
			UDM		USDM			UDEDM					
	L [nm]	L [nm]	L [nm]	ε [10^{-3}]	L [nm]	σ [GPa]	ε [10^{-3}]	L [nm]	σ [GPa]	ε [10^{-3}]	u [MJ/m3]	L [nm]	ε [10^{-3}]
Low-Range	7.89	9.32	11.45	3.89	14.14	0.56	6.25	12.51	0.44	4.92	1.08	9.90	3.42
Whole-Range	6.39	10.09	1190	12.6	-53.5	1.48	100.3	-47.1	1.51	14.3	10.7	11.9	5.66

References

1. Nath, D.; Singh, F.; Das, R., X-ray diffraction analysis by Williamson-Hall, Halder-Wagner and size-strain plot methods of CdSe nanoparticles-a comparative study. *Mater Chem Phys* **2020,** 239:122021.
2. Delhez, R.; de Keijser, T. H.; Mittemeijer, E. J., Determination of Crystallite Size and Lattice Distortions through X-Ray Diffraction Line Profile Analysis. *Fresenius Z Anal Chem* **1982,** 312, 1-16.
3. Yogamalar, R.; Srinivasan, R.; Vinu, A.; Ariga, K.; Bose, A. C., X-ray peak broadening analysis in ZnO nanoparticles. *Solid State Commun* **2009,** 149, (43-44), 1919-1923.
4. Speziale, S.; Duffy, T. S., Single-crystal elastic constants of fluorite (CaF_2) to 9.3 GPa. *Phy Chem Minerals* **2002,** 29, (7), 465-472.
5. Dey, P. C.; Das, R., Effect of silver doping on the elastic properties of CdS nanoparticles. *Indian J Phys* **2018,** 92, (9), 1099-1108.
6. Motevalizadeh, L.; Heidary, Z.; Ebrahimizadeh Abrishami, M., Facile template-free hydrothermal synthesis and microstrain measurement of ZnO nanorods. *Bull. Mater. Sci.* **20014,** 37, 397-405.
7. Taurozzi, J. S.; Hackley, V. A.; Wiesner, M. R., Ultrasonic dispersion of nanoparticles for environmental, health and safety assessment - issues and recommendations. *Nanotoxicology* **2011,** 5, (4), 711-29.
8. Robertson, J. D.; Rizzello, L.; Avila-Olias, M.; Gaitzsch, J.; Contini, C.; Magon, M. S.; Renshaw, S. A.; Battaglia, G., Purification of Nanoparticles by Size and Shape. *Sci Rep* **2016,** 6:27494.
9. Molaiyan, P.; Witter, R., Surface defect-enhanced conductivity of calcium fluoride for electrochemical applications. *Mat Design Process Comm* **2019,** 1: e44.
